# Characterization and phylogenetic analysis of the complete mitochondrial genome of the rainbow krib, *Pelvicachromis pulcher* (Perciformes: Cichlidae)

**DOI:** 10.1080/23802359.2022.2079099

**Published:** 2022-06-02

**Authors:** Sang-Eun Nam, Hye-Jin Eom, Hyoung Sook Park, Jae-Sung Rhee

**Affiliations:** a Department of Marine Science, College of Natural Sciences, Incheon National University, Incheon, South Korea; b Department of Song-Do Bio-Environmental Engineering, Incheon Jaeneung University, Incheon, South Korea; c Research Institute of Basic Sciences, Incheon National University, Incheon, South Korea; d Yellow Sea Research Institute, Incheon, South Korea

**Keywords:** Complete mitogenome, cichlid fish, *Pelvicachromis pulcher*, phylogenetic analysis

## Abstract

We report the complete mitochondrial genome information of the rainbow krib, *Pelvicachromis pulcher* (Boulenger 1901). Illumina HiSeq genome sequencing allowed the assembly of a circular mitogenome of 17,196 base pairs (bp) from *P. pulcher* consisting of 47% GC nucleotides, 13 protein-coding genes (PCGs), 2 ribosomal RNA (rRNA) genes, 22 transfer RNA (tRNA) genes, and a putative control region in the typical teleost gene composition. The gene order of the *P. pulcher* mitogenome was identical to that of other cichlid species. A maximum likelihood phylogenetic tree based on mitochondrial PCGs showed a relationship of *P. pulcher* with a cichlid *Tylochromis polylepis* (Boulenger 1900), suggesting that more complete mitogenomes are needed to explore mitogenome evolution in West African tribes and riverine cichlids, as this genomic information is the first complete mitogenome in the tribe Chromidotilapiini.

The family Cichlidae is one of the most species-rich clades in spiny-rayed fish ( acanthomorphs; Kornfield and Smith 2000; Nelson et al. 2016), with a wide distribution range, mainly in Africa, South America, and Middle America, and with abundance and ecological diversity (Smith et al. 2008). Among cichlid fish, clades of African cichlid (Pseudocrenilabrinae) have major adaptive radiations in East African lakes (i.e. Lake Tanganyika, Lakes Victoria, and Lake Malawi) with different adaptive responses and phenotypes (Muschick et al. 2012; Brawand et al. 2014) and are sister to monophyletic Neotropical cichlids (Cichlinae) (Sparks and Smith 2004). In recent decades, the evolutionary and phylogenetic relationships among East African tribes have been clarified (Schwarzer et al. 2009; Brawand et al. 2014; Meyer et al. 2015; Irisarri et al. 2018; Schedel et al. 2019). Although West African cichlid tribes form the most basal African taxa (Farias et al. 2001), little is known about the diversity and phylogenetic evolution of West African tribes and riverine cichlids (e.g. Chromidotilapiini, Coptodonini, Hemichromini, Pelmatochromini, Tylochromini).

The rainbow krib, *Pelvicachromis pulcher* (Boulenge 1901) belongs to the tribe Chromidotilapiini, also known as West African cichlid, dwarf African cichlid, or kribensis. The fish are endemic to the African freshwaters of southern Nigeria, western Cameroon, and eastern Benin. The rainbow krib is a popular ornamental species because of its small size (maximum length of adults of approximately 13 cm), ease of handling, a variety of color morphs, peaceful behavior the in aquaria, sexual dimorphism, relatively simple breeding process, and activity in brood care as bi-parental species. The latter is recognized by the different behaviors of females for offspring care after cave spawning (speleophils) territorial defense behavior of males near the cave (Martin and Taborsky 1997). There is no information on the complete mitogenome in the tribe Chromidotilapiini. Incomplete mitochondrial PCGs and genomic markers (e.g. *COI*, *16S rRNA*, and *histone H3*) of *P. pulcher* have been registered in the National Center for Biotechnology Information GenBank database. As many complete mitogenomes have been published in East African cichlids, complete mitogenome information of *P. pulcher* can provide an essential resource to infer geographical distribution, phylogenetic relationships, and evolutionary history of Chromidotilapiini.

A specimen of *P. pulcher* was collected from the River Sombreiro (5°09′N, 6°43′E), Rivers State, Nigeria. The specimen and DNA were deposited at the Research Institute of Basic Sciences of Incheon National University (Specimen ID: 2013-Cichlidae-08; https://www.inu.ac.kr/user/indexMain.do?siteId=ribs) by Dr. Sang-Eun Nam (se_nam2@inu.ac.kr). Genomic DNA was prepared from a muscle sample using the DNeasy Blood and Tissue kit (Qiagen, Hilden, Germany) according to the manufacturer’s standard protocol. A fragment library was prepared using the TruSeq DNA Sample Preparation Kit (Illumina, San Diego, CA, USA) as previously described (Nam and Rhee 2020) prior to Illumina HiSeq sequencing. The sequencing library was prepared by random fragmentation of the DNA sample, followed by 5′ and 3′ adapter ligation. Raw reads were obtained from the sample that passed the quality control check on the Illumina HiSeq platform at Macrogen, Inc. (Seoul, South Korea). Adapter sequences, low quality reads, reads with >10% unknown bases, and ambiguous bases were removed to obtain high quality assembly. After the quality check process, 28,918,638 filtered reads were obtained from 39,752,230 raw reads. Subsequently, *de novo* assembly was conducted with various k-mers using SPAdes (Bankevich et al. 2012). A circular contig of the *P. pulcher* mitogenome was obtained. The resulting contig consensus sequence was annotated using MITOS2 (Bernt et al. 2013) and tRNAscan-SE 2.0 (Lowe and Eddy 1997). BLAST searches confirmed the identity of the genes (http://blast.ncbi.nlm.nih.gov).

The nucleotide composition of the *P. pulcher* circular 17,196 bp mitogenome (GenBank accession no. MZ357707) was 26.4% A, 30.6% C, 16.3% G, and 26.7% T. The gene order and composition of the *P. pulcher* mitogenome were identical to those of other mitogenomes of cichlids. A phylogenetic tree was constructed using the concatenated set of all 13 PCGs of the *P. pulcher* mitogenome, 30 published complete mitogenomes of cichlids, and an outgroup from the family Balistidae (Figure 1). JModelTest ver. 2.1.10 (Darriba et al. 2012) was used to select the best substitution model and the HKY + G + I substitution model was applied to perform a maximum-likelihood (ML) analysis using PhyML 2.4.5 (Guindon and Gascuel 2003) with 1000 bootstrap replicates. The overall topology of each tribe was consistent with previous phylogenetic results (Schwarzer et al. 2009; Irisarri et al. 2018). Although the *P. pulcher* mitogenome formed a sister group with the mitogenome of *Tylochromis polylepis* with strong support, phylogenetic analysis should be improved by an incorporation of additional complete mitogenomes of basal African taxa, as there are very few full mitogenome sequences available for Africa cichlids outside of tribes Haplochromini and Oreochromini.

**Figure 1.**
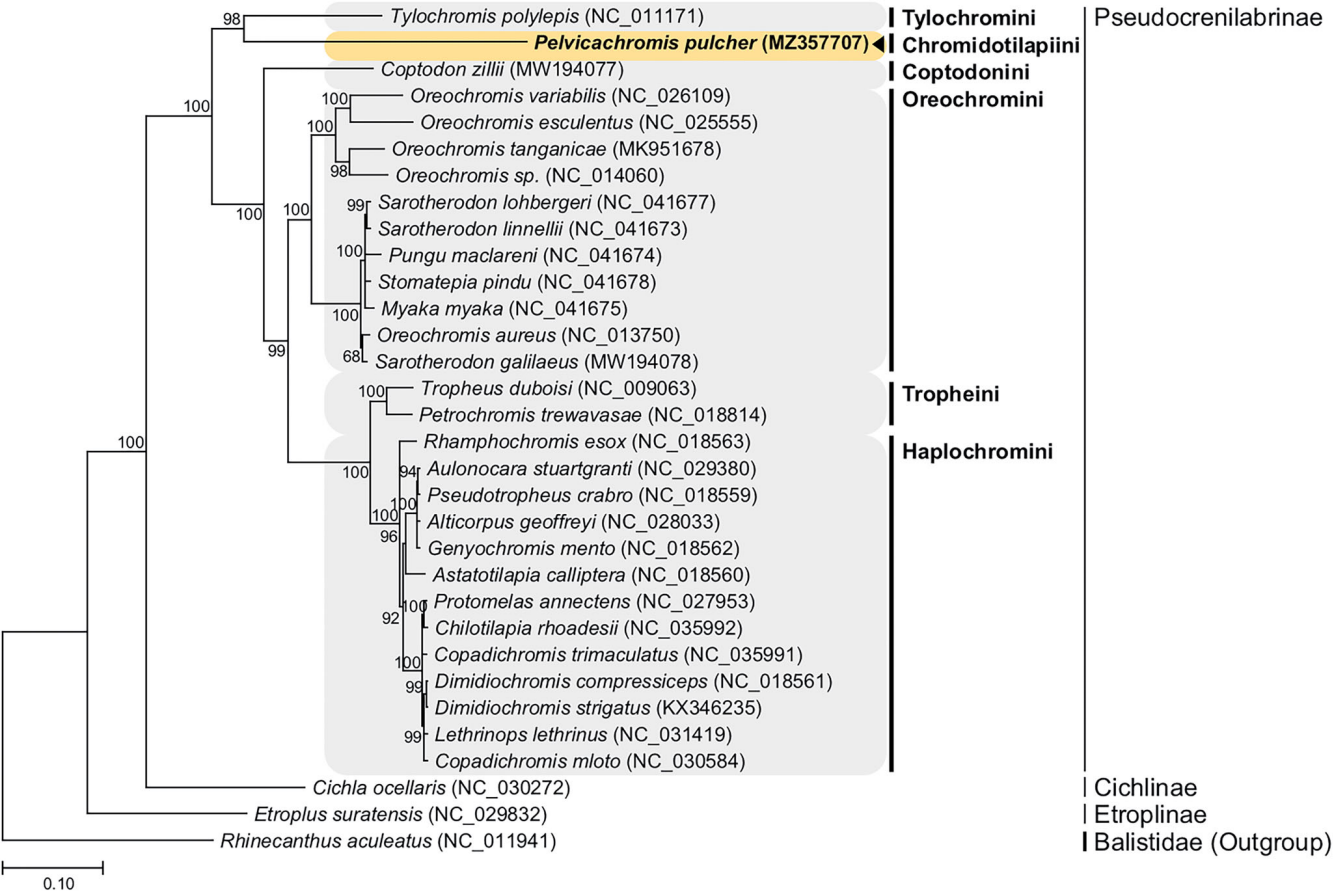
Maximum-likelihood (ML) phylogeny of 30 published complete mitogenomes of cichlids and an outgroup from the family Balistidae based on the concatenated nucleotide sequences of protein-coding genes (PCGs). The phylogenetic analysis was performed using the maximum likelihood method and GTR + G + I model with a bootstrap of 1000 replicates. Numbers on the branches indicate ML bootstrap percentages. DDBJ/EMBL/Genbank accession numbers for published sequences are incorporated. The black triangle indicates the cichlid analyzed in this study.

## Data Availability

BioProject, BioSample, and SRA accession numbers are https://www.ncbi.nlm.ni h.gov/bioproject/PRJNA743752, https://www.ncbi.nlm.nih.gov/biosample/SAMN20059975, and https://www.ncbi.nlm.nih.gov/sra/?term=SRR15348982, respectively. The data that support the findings of this study are available at the National Center for Biotechnology Information (NCBI) at https://www.ncbi.nlm.nih.gov, with the accession number MZ357707.
